# Regulation of Extracellular Matrix Organization by BMP Signaling in *Caenorhabditis elegans*

**DOI:** 10.1371/journal.pone.0101929

**Published:** 2014-07-11

**Authors:** Robbie D. Schultz, Emily E. Bennett, E. Ann Ellis, Tina L. Gumienny

**Affiliations:** 1 Department of Molecular and Cellular Medicine, Texas A&M Health Science Center College of Medicine, College Station, Texas, United States of America; 2 Interdisciplinary Program in Genetics, Texas A&M University, College Station, Texas, United States of America; 3 Microscopy & Imaging Center, Texas A&M University, College Station, Texas, United States of America; National Institute of Biological Sciences, Beijing, China

## Abstract

In mammals, Bone Morphogenetic Protein (BMP) pathway signaling is important for the growth and homeostasis of extracellular matrix, including basement membrane remodeling, scarring, and bone growth. A conserved BMP member in *Caenorhabditis elegans,* DBL-1, regulates body length in a dose-sensitive manner. Loss of DBL-1 pathway signaling also results in increased anesthetic sensitivity. However, the physiological basis of these pleiotropic phenotypes is largely unknown. We created a DBL-1 over-expressing strain and show that sensitivity to anesthetics is inversely related to the dose of DBL-1. Using pharmacological, genetic analyses, and a novel dye permeability assay for live, microwave-treated animals, we confirm that DBL-1 is required for the barrier function of the cuticle, a specialized extracellular matrix. We show that DBL-1 signaling is required to prevent animals from forming tail-entangled aggregates in liquid. Stripping lipids off the surface of wild-type animals recapitulates this phenotype. Finally, we find that DBL-1 signaling affects ultrastructure of the nematode cuticle in a dose-dependent manner, as surface lipid content and cuticular organization are disrupted in animals with genetically altered DBL-1 levels. We propose that the lipid layer coating the nematode cuticle normally prevents tail entanglement, and that reduction of this layer by loss of DBL-1 signaling promotes aggregation. This work provides a physiological mechanism that unites the DBL-1 signaling pathway roles of not only body size regulation and drug responsiveness, but also the novel Hoechst 33342 staining and aggregation phenotypes, through barrier function, content, and organization of the cuticle.

## Introduction

Intercellular signaling by bone morphogenetic proteins (BMPs), members of the Transforming Growth Factor-β (TGF-β) superfamily of signaling morphogens, is critical for a variety of normal developmental and homeostatic processes, including extracellular matrix deposition and remodeling. BMPs are critical for proper limb outgrowth during development [1]. Recombinant human BMPs are used clinically to repair and replace bone [2]. Perturbation of BMP signaling levels can contribute to pathogenic conditions including bone disorders and cancers [3].

The invertebrate *Caenorhabditis elegans* is an established genetic model system for studying BMP signaling. BMP member DBL-1 (Drosophila Dpp and BMP-like-1) regulates post-embryonic body size and other phenotypes [4]–[6]. Animals with increased DBL-1 signaling are longer than wild-type animals, while loss of signaling results in smaller animals (Table 1). The body length phenotype develops during postembryonic development, and is not based on cell number, as this eutelic species has a fixed somatic cell number among its members [6]–[8]. Studies to address how DBL-1 signaling regulates body size has revealed a canonical BMP signaling pathway exists to transmit the secreted DBL-1 signal from the cell membrane through a set of conserved receptors to the nucleus by Smad transcriptional regulators [9]. The cellular focus of the body size phenotype is the hypodermis, an epidermal tissue that surrounds the animal's internal tissues and synthesizes the nematode cuticle, a sturdy, protective extracellular matrix [10]–[12]. The DBL-1 receptors, Smads, other regulatory factors, and a multitude of pathway targets are expressed in this tissue. However, the cellular mechanisms underlying the body size phenotype of this molecular pathway remain unclear. Previous work to address the question of how DBL-1 regulates body size has provided evidence of a partial contribution by endoreduplication within these hypodermal cells [7], [8], [11], [13]–[15]. Multiple studies show that expression of a number of transcriptional targets, including cuticular components, is altered by changes in DBL-1 signaling [16]–[19]. Loss of single cuticular proteins can also alter nematode body length [16], [20], [21].

**Table 1:** DBL-1 is a dose-dependent regulator of body length and annular width.

Genotype	Body length	P-value	n	Annular width	P-value	n
Wild type	100±2	-	30	100±4	-	33
*dbl-1(nk3)*	79±2	<0.0001^A^	30	82±3	<0.0001^A^	32
*dbl-1(nk3); dbl-1(++)*	97±2	<0.0001^B^	30	N.D.	-	-
*dbl-1(++)*	110±2	<0.0001^A^	30	112±4	<0.0001^A^	30
*lon-2(e678)*	119±2	<0.0001^A^	29	N.D.	-	-

Body length and annular width represent average measurements relative to wild type ± the 95% confidence interval. P-values compare data to wild type (^A^) or *dbl-1(nk3)* (^B^) using the unpaired t-test. N.D. is not determined.

Loss of DBL-1 signaling increases sensitivity to different drug types in *C. elegans* [22], [23]. While mutation of drug target genes can affect drug response of the animal, a compromised nematode permeability barrier is also associated with increased anesthetic sensitivity [24], [25]. While it has been proposed that DBL-1 also affects drug entry, rather than affecting the function of the drug targets themselves, the basis of this DBL-1 function is unresolved [22].

Do the nematode BMP-mediated phenotypes have a cellular basis in extracellular matrix deposition and patterning, like many mammalian BMP phenotypes? Based on the observations that (1) the cuticle-secreting tissue contributes to the DBL-1-mediated body size phenotype [11], [12], [15], (2) cuticle-specific genes are highly regulated by DBL-1 signaling [11], [17], [19], and (3) changes in cuticle can affect both body morphology and drug response [21], [24], [25], we hypothesized that these apparently unrelated DBL-1-mediated phenotypes are consequences of altered cuticle. We asked if the cuticle was affected in nematodes with genetically manipulated levels of DBL-1 signaling. Animals overexpressing tagged DBL-1 are more resistant to drugs, showing a dose-dependent response to anesthetics by DBL-1. Using a novel microwave-based permeability assay for live animals and by genetically disrupting cross-linkages within the cuticle, we show that DBL-1 regulates cuticular barrier function. This physiological change in cuticular permeability is linked to the drug response phenotype displayed by DBL-1 pathway mutant animals. Loss of DBL-1 also permits tails to become entangled, forming “worm-stars”. This oriented aggregation is phenocopied in wild-type animals that have had their surface coat and lipids stripped, indicating that this phenotype in *dbl-1* mutant animals is also cause by altered surface properties. Through ultrastructure studies, we identified a correlation of DBL-1 signaling level with substantial changes of cuticular layer organization and surface lipid amount.

We propose that a common physiological mechanism, alteration of the cuticle, largely explains both the body length and drug response phenotypes, and underlies the worm-star aggregation defect we identified in *dbl-1* loss-of-function populations. Furthermore, this work shows that BMP pathway signaling, which in mammals affects bone and other extracellular matrix growth and remodeling processes, also affects extracellular matrix in the invertebrate *C. elegans*, revealing a conserved function for the BMP family of cell signaling molecules.

## Materials and Methods

### Strains and maintenance

*C. elegans* strains used in these studies were derived from the wild-type variety Bristol strain N2 and were cultured on nematode growth media (NGM) plates as previously described [20]. All strains were cultured on *E. coli* strain OP50 at 20°C, except where noted. Strains used include: N2, TLG634 *sma-3(wk30)* III; *him-5(e1490)* V, TLG182 *texIs100 [dbl-1p::dbl-1:gfp + ttx-3p::rpf]* IV (referred to as *dbl-1(++)* in this paper), TLG269 *texIs100* IV; *dbl-1(nk3)* V, TP12 *kaIs12 [col-19p::col-19:gfp]*, which we discovered appears to be linked to *texIs100* on chromosome IV [26], NU3 *dbl-1(nk3)* V, CL261 *him-5(e1940)* V; *srf-5(ct115)* X [27], and CB678 *lon-2(e678)* X.

We sequenced the *dbl-1(nk3)* lesion (previously called *cet-1(kk3)* [4]) and identified a 5595 bp deletion that removes the 5′ untranslated region and all but 33 bp of *dbl-1* 3′ coding sequence. The flanking sequence is CTGCGCCTCC. GACATGCGGG.

### Molecular biology

To generate a clone with *egfp* fused to the mature *dbl-1* sequence downstream of the *dbl-1* prodomain, we first used a sequential PCR fusion technique using cosmid T25F10 as template for the *dbl-1* reactions [28]. Using the Gateway recombinational cloning system [29], we then inserted the PCR product into a Gateway donor vector with BP Clonase II (Life Technologies, Grand Island, NY) to generate *gfp:dbl-1* in pDONR221. Next, we PCR amplified and inserted 2 kb sequence 5′ to the start site of *dbl-1* into pDONRP4-P1R. Finally, we performed a multisite Gateway reaction between the *gfp:dbl-1* in pDONR221, the *dbl-1* promoter in pDONRP4-P1R, and the pDEST6-R4-R2 destination vector (gift of I. Hope, University of Leeds, Leeds, UK) using LR Clonase II Plus (Life Technologies, Grand Island, NY) [30]. The resulting construct contained *dbl-1p::gfp:dbl-1* and was named pK13-1.2. Primers used are available upon request.

### Generation of transgenic strains

Initial germline transformation of nematode strains with plasmid DNAs was performed by microinjection [31]. The *gfp*-tagged *dbl-1* construct pK13-1.2 was injected into N2 at 50 ng/µL with 50 ng/µL co-injection marker *ttx-3p::rfp*, which is expressed in the two AIY head neurons (gift of O. Hobert, Columbia University Medical Center, New York, NY, and C. Rongo, Rutgers University, Piscataway, NJ) [32]. We then created an integrated transgene from a strain expressing GFP-tagged DBL-1. Low copy integrated lines (generously created for us by B. Grant, Rutgers University, Piscataway, NJ) did not express visible levels of tagged DBL-1, nor did they affect body size (data not shown). UV/TMP integration of a multicopy extrachromosomal array yielded *texIs100* and four other alleles, which were backcrossed five times to remove extraneously UV/TMP-induced mutations and mapped. *texIs100* was selected for further studies based on its relatively high expression level, activity (body length phenotype), and location (chromosome IV).

### Body length measurements

Body measurements of animals were performed as previously described [33]. Specifically, about 30 staged young adult animals were transferred to 2% agar pads on glass slides and were imaged when moving forward at 60× magnification using iVision-Mac software (BioVision Technologies, Exton, PA) and a Retiga-2000R CCD camera (QImaging Corporation, Surrey, BC, Canada) mounted on a Nikon SMZ1500 dissecting microscope (Nikon Instruments, Inc., Melville, NY).

Lengths of animals were determined using the length measurement image tool within iVision-Mac software (BioVision Technologies, Exton, PA). Average body length values of strain populations were converted to percent wild-type average body lengths using staged wild-type control populations that were imaged the same day as the experimental strain(s). 95% confidence intervals were calculated using Prism (GraphPad Software, Inc., La Jolla, CA). P-values (using the unpaired t-test) were determined using Excel (Microsoft Corporation, Redmond, WA).

### Drug sensitivity assays

Sensitivity to drugs was assayed as previous described [34]. Briefly, about 40 staged young adult animals were transferred to NGM plates containing 0–1 mM levamisole HCl, 0.2% (wt/vol) tricaine (ethyl 3-aminobenzoate methanesulfonate), 0.2% (vol/vol) IP2P (1-phenoxy-2-propanol), or 1 mM sodium azide. For standard drug sensitivity assays, the number of animals moving was scored by visual inspection every 15 minutes and was defined as response (movement) to prodding. For levamisole dose-curve assays, the number of animals moving was scored by visual inspection at 60 minutes and was defined as response (movement) to prodding. Three independent trials were performed and the results were pooled, with at least 117 animals total for each genotype at each time point. The average fraction of animals moving, standard error of the mean (SEM), p-values (using the unpaired t-test), and EC_50_ (by log transformation) were determined using Excel (Microsoft Corporation, Redmond, WA).

### RNA interference

RNA interference (RNAi) was performed as previously described [35], with the exception that generations of animals were continuously grown on IPTG-containing NGM plates that were seeded with bacteria expressing gene-specific double stranded RNA. Briefly, single colonies of HT115 bacteria containing relevant plasmids (Thermo Fisher Scientific, Waltham, MA) were selected, isolated, and grown overnight in carbenicillin, then induced for 4 to 5 hours with IPTG to express double stranded RNA from the plasmid. Each bacterial growth was spotted onto NGM plates containing carbenicillin and IPTG and dried. Animals were then transferred to and continuously cultured on NGM plates seeded with RNAi bacterial lawns at 15°C for use in either the drug sensitivity assays or fluorescent microscopy and imaging. Drug sensitivity scoring was performed as described above. Imaging was performed as described below.

### Hoechst 33342 staining and quantification

To provide more consistent staining, L3 animals were staged by allowing gravid adults to lay embryos for about 16 hours on a plate. These animals that had never been starved or bleached were then washed in M9 buffer three times to remove residual bacteria. Next, animals were stained with the cuticle impermeable dye, Hoechst 33342 (2′-[4-ethoxyphenyl]-5-[4-methyl-1-piperazinyl]-2,5′-bi-1H-benzimidazole trihydrochloride trihydrate, Life Technologies, Grand Island, NY) by microwave irradiation treatment. Using the PELCO BioWave microwave (Ted Pella, Redding, CA), live animals were stained by microwaving at 20°C with intermittent vacuum at 200 watts (W) for a 6-minute cycle (2 minutes on, 2 minutes off, 2 minutes on) in 1 µg/ml Hoechst 33342 in M9 buffer. Prior to imaging, animals were washed four times in M9 buffer. Imaging was performed as described below.

The number of animals displaying fluorescently stained hypodermal nuclei was scored by visual inspection. Three independent trials were performed and the results were pooled, with at least 218 animals per trial for each genotype. The average fraction of stained animals, SEM, and p-values (using the unpaired t-test) were determined using Excel (Microsoft Corporation, Redmond, WA).

### Worm-star formation assays and imaging

Staged adult animals were washed in M9 buffer three times to remove residual bacteria. Animals were then incubated for three hours at room temperature in 7.5 ml M9 buffer (without OP50 bacteria) in 60 mm petri dishes tilted at a slight angle to concentrate animals in a single area of the plate. The number of animals in worm-star aggregations, clusters of two or more animals entangled at their tails, was quantified for each genotype by visual inspection using a dissecting microscope. Three independent trials were performed and results were pooled, with approximately 150 to 400 animals per trial for each genotype.

To assay the effect of extracting surface lipids in wild-type populations, mixed stage animals that had been washed in M9 buffer three times to remove residual bacteria were briefly washed in 30% ethanol in M9 buffer. Animals were washed three additional times in M9 buffer to remove all traces of ethanol. Animals were then incubated overnight at room temperature in 7.5 ml M9 buffer in 60 mm petri dishes tilted at a slight angle to concentrate animals in a single area of the plate. The number of animals in worm-star aggregations was quantified by visual inspection using a dissecting microscope. Four independent trials were performed and results were pooled, with at least 150 animals per trial for each genotype. Worm-stars were imaged using the same imaging system used for body length measurements.

### Annuli staining and measurements

The cuticle of staged adult animals was stained with DiI (1,1′-dioctadecyl-3,3,3′,3′-tetramethylindocarbocyanine perchlorate, Biotium Inc., Hayward, CA) as previously described [36]. Specifically, staged young adult animals were washed once with M9 buffer with 0.5% (vol/vol) Triton X-100, then two times in M9 buffer. Next, animals were stained in 30 µg/ml DiI in M9 for approximately three hours while shaking at high speed at 20°C. Prior to imaging, animals were washed in M9 buffer to remove residual DiI. Confocal imaging was performed as described below.

Annuli widths were determined using the length measurement image tool within iVision-Mac. The width of ten annuli was measured for each animal to compensate for any minor deviations in individual annuli size. Average width values from each strain were converted to percent wild-type average widths using staged wild-type control populations that were imaged the same day as the experimental strains. 95% confidence intervals were calculated using Prism (GraphPad Software, Inc., La Jolla, CA). P-values (using the unpaired t-test) were determined using Excel (Microsoft Corporation, Redmond, WA).

### Wheat germ agglutinin staining

Lectin staining was performed as previous described [37]. Briefly, populations of staged adult animals were washed three times in M9 buffer to remove any residual bacteria. To further remove any residual bacteria from the cuticular surface, animals were incubated in M9 buffer for an hour while gently shaking at 20°C. Animals were then stained in 200 µg/ml rhodamine-conjugated wheat germ agglutinin (WGA) (Vector Laboratories, Inc., Burlingame, CA) in M9 buffer for one hour while gently shaking at 20°C. Prior to imaging, animals were washed four times in M9 buffer. Imaging was performed as described below.

### Confocal microscopy and imaging

Animals were immobilized in 2.5% (wt/vol) 0.1 µm diameter polystyrene beads (00876-15, Polysciences Inc., Warrington, PA) in 1 mM levamisole on 10% agarose pads [38]. Fluorescent images were acquired on a Retiga-SRV CCD camera (Quantitative Imaging Corporation, Surrey, BC, Canada) mounted on a BD Carv II spinning disk confocal (BD Biosystems, San Jose, CA) on a Zeiss A1 compound microscope base (Carl Zeiss, Inc., Jena, Germany) fitted with GFP HQ, Rhodamine HQ, and DAPI filters. 10x/0.3 NA Plan-Neofluar and 63x/1.4 NA oil Plan-Apochromat objectives (Carl Zeiss, Inc., Jena, Germany) and iVision-Mac software (BioVision Technologies, Exton, PA) were used for image acquisition.

### Transmission electron microscopy and imaging

As the cuticle of *C. elegans* is extremely tough, we optimized methods for transmission electron microscopy (TEM) sample preparation using the PELCO BioWave microwave with ColdSpot technology (Ted Pella, Redding, CA). For typical bench-top methods, the cuticle of each nematode is sliced during fixation to allow the fixative past the impenetrable cuticle [39]. Consistent with previous findings [40], we found that treatment with microwave irradiation allows the fixative and other treatments to penetrate the cuticle and infiltrate the tissue, eliminating any need to cut the cuticle.

Adult animals were washed in M9 buffer two times to remove residual bacteria. Next, animals were immersed in fixative (2.5% glutaraldehyde, 2% paraformaldehyde, and 0.1% (wt/vol) malachite green in working buffer (0.1 M HEPES, pH 7.4, containing 2 mM MgCl_2_)). Malachite green was included as an additive to the primary fixative to preserve and stain lipids that are normally stripped from specimens during standard TEM fixation procedures [41]. For all microwave steps, specimens were microwaved at 20°C with a 37°C cut-out temperature and intermittent vacuum at 250 W unless otherwise noted. While immersed in fixative, animals were microwaved for an initial 6-minute cycle (2 minutes on, 2 minutes off, 2 minutes on), incubated at room temperature for about an hour, then microwaved again for another 6-minute cycle (2 minutes on, 2 minutes off, 2 minutes on). Animals were then washed three times in working buffer, microwaving each wash for one minute. To provide more contrast, specimens were post-fixed in 1% (wt/vol) osmium tetroxide with 1.5% (wt/vol) potassium ferricyanide in working buffer for 15 minutes at room temperature and then microwaved at 100 W for one minute. Samples were then washed two times in water, microwaving each wash one minute. Animals were next stained *en bloc* with 0.5% aqueous uranyl acetate at room temperature over night. Specimens were washed in water one time with a one-minute microwave cycle. The samples were then dehydrated through a graded methanol series, starting at 5% (vol/vol) and continuing to 100% (vol/vol) methanol at 5% intervals, microwaving for one minute at each grade. Finally, animals were suspended in propylene oxide, infiltrated (each infiltration step was accompanied by a 6-minute microwave cycle (2 minutes on, 2 minutes off, 2 minutes on)), and embedded in a Quetol 651-modified Spurr low viscosity epoxy resin [42]. Transverse gold sections were taken from the midsection of young adult hermaphrodites and post-stained with 2% (wt/vol) aqueous uranyl acetate followed by Reynolds lead citrate [43]. At least six animals of each genotype were examined and photographed on a JEOL 1200EX transmission electron microscope (JEOL Ltd., Tokyo, Japan) at an accelerating voltage of 100 kV.

### TEM measurements

The depth and length of cuticular components were determined from micrographs of cross-sections using the tape measure tool within SIA Micrograph MaxIm DL5 software (Diffraction Limited, Ottawa, Canada). Components of at least six animals were measured per genotype. Averages of depth or length, SEM, and p-values (using the unpaired t-test) were determined using Excel (Microsoft Corporation, Redmond, WA).

## Results and Discussion

### GFP-tagged DBL-1 is functional

**Figure 1:**
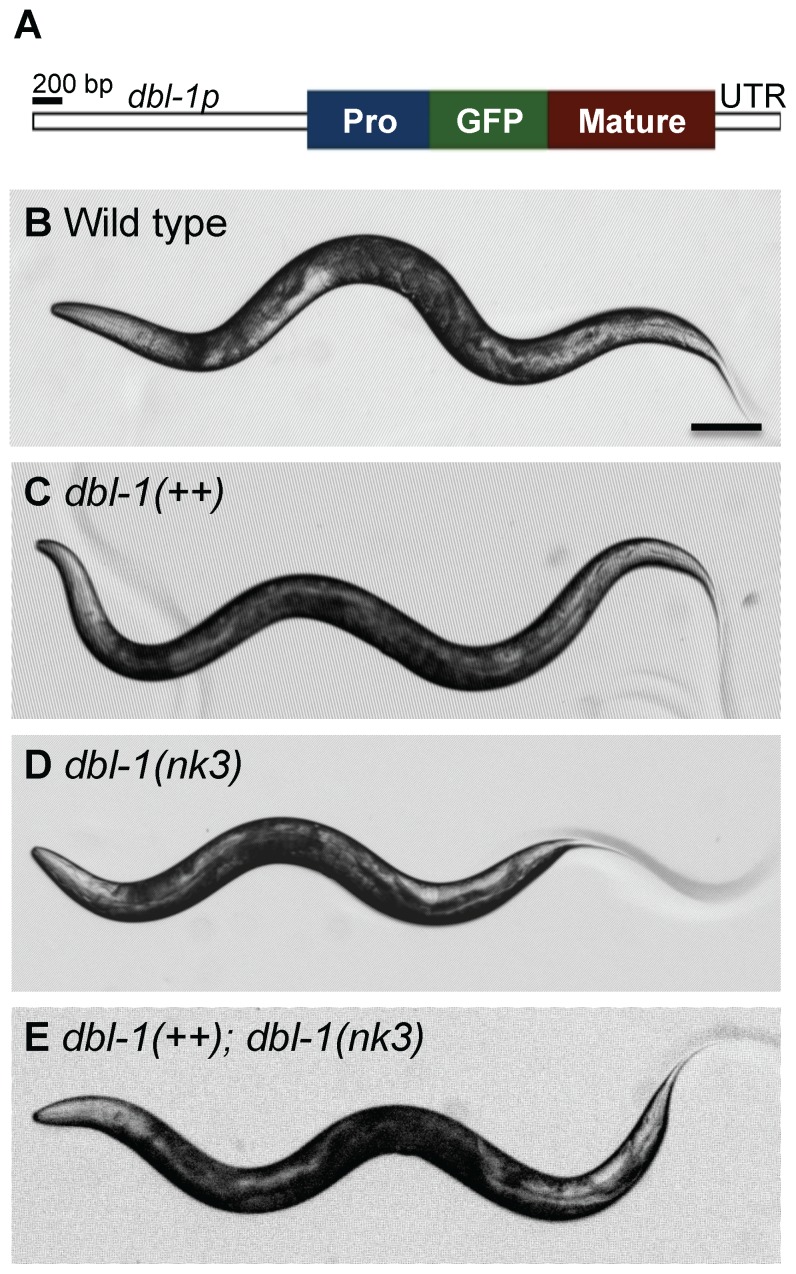
GFP-tagged DBL-1 is bioactive. (A) Schematic diagram of GFP-tagged DBL-1 expressed from the *dbl-1* promoter (*dbl-1p*). The GFP-tag (green) is inserted downstream of the prodomain (blue) and upstream of the DBL-1 mature domain (red). The construct also contains the *dbl-1* specific 3′ untranslated region (UTR). (B–E) Body lengths of wild-type (B), *dbl-1(++)* (C), *dbl-1(nk3)* (D), and *dbl-1(nk3)* mutant animals expressing the GFP-tagged DBL-1 transgene (E). Scale bar  =  100 µm.

We made a strain expressing transgenic GFP-tagged DBL-1 (allele name *texIs100*) and integrated this transgene into in a wild-type background. As these animals express both endogenous DBL-1 and the GFP-tagged DBL-1, we call this overexpressing strain *dbl-1(++)*. The transgene is expressed from 2 kb of sequence upstream of the *dbl-1* initiator codon, which expresses in cholinergic motor neurons previously identified to express DBL-1 (Figure 1A and data not shown) [4], [6]. DBL-1 expressed from the *texIs100* transgene is bioactive, as transgenic animals in a wild-type background are longer than normal, and animals lacking endogenous *dbl-1* product are restored to a more normal body length with *texIs100* (Table 1 and Figure 1B-E). Fluorescence analyses will be described elsewhere.

### Increased DBL-1 pathway signaling results in drug resistance

**Figure 2:**
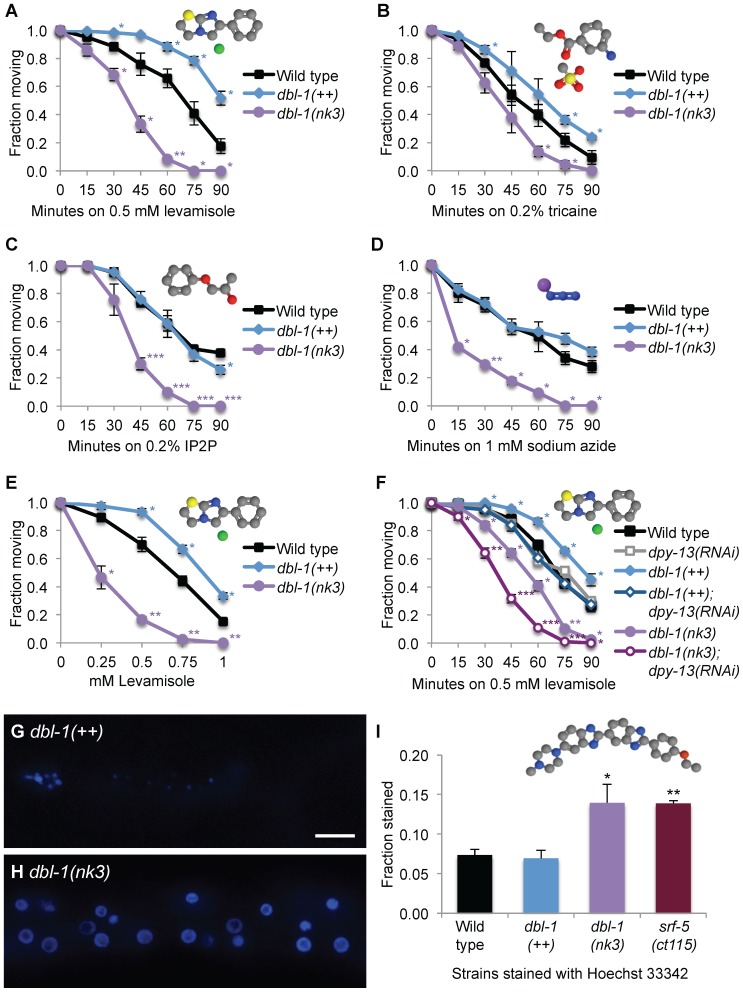
DBL-1 regulates cuticular permeability. (A-D) Sensitivity to levamisole (A), tricaine (B), IP2P (C), and sodium azide (D) was measured over time in animals with wild-type, increased (*dbl-1(++)*), and reduced (*dbl-1(nk3)*) DBL-1 pathway signaling. (E) Sensitivity to different levamisole doses by animals with wild-type, reduced (*dbl-1(nk3)*), and increased (*dbl-1(++)*) DBL-1 pathway signaling. (F) Sensitivity to levamisole in animals with different DBL-1 levels treated with *C06C3.5* (pseudogene control) or *dpy-13* RNAi was measured over time. (G–I) Hoechst 33342 staining in *dbl-1(++)* (G) or *dbl-1(nk3)* (H) animals. Faint intestinal autofluorescence is visible in (G). Scale bar = 10 µm. The fraction of animals that stain with Hoechst 33342 is shown in (I). Error bars indicate the mean ± SEM. P-values compare data to wild type (***P≤0.0001; **P≤0.001; *P≤0.05) using the unpaired t-test. Chemical structures were drawn using Jmol: an open-source Java viewer for chemical structures in 3D. http://www.jmol.org/. Molecule key: gray, carbon; blue, nitrogen; purple, sodium; green, chloride; yellow, sulfur; and red, oxygen.

Animals deficient in DBL-1 signaling are hypersensitive to the anesthetizing effects of the cholinergic agonists levamisole and nicotine and the cholinesterase inhibitor aldicarb, which prevents breakdown of acetylcholine [22], [23]. Confirming these results, we also found that *dbl-1(nk3)* mutant animals are hypersensitive to levamisole-induced paralysis (Figure 2A). To test if this response was DBL-1 dose-dependent, we assayed paralysis in animals with increased DBL-1 signaling levels. After incubating animals on plates containing 0.5 mM levamisole for 75 minutes, over half of wild-type animals were immobilized and all *dbl-1(nk3)* animals were paralyzed (Figure 2A). However, only 20% of the *dbl-1(++)* population was anesthetized (Figure 2A). To confirm this levamisole resistance phenotype is specific to increased DBL-1 pathway signaling, we also tested animals lacking the DBL-1 negative regulator LON-2. *lon-2(e678)* mutants showed results similar to overexpressing the DBL-1 ligand (data not shown). These results reveal animals with increased DBL-1 signaling display resistance to levamisole-induced paralysis, showing that DBL-1 is a dose-dependent regulator of levamisole response.

We reasoned the altered sensitivity to levamisole in the DBL-1 variant backgrounds could be caused by specific modulation of acetylcholine receptor signaling pathway or by altered accessibility of the drug to its receptors. To distinguish between these models and investigate the specificity of altered drug sensitivity due to dose of DBL-1, we analyzed the effects of three additional nematode anesthetics with different modes of action and molecular weights. We tested tricaine, which has a higher molecular weight of 261 as compared to 241 for levamisole. Further distinguishing the two anesthetics, tricaine suppresses the nervous system by reducing transmission of action potential of the nerve [44]. Using 0.2% tricaine, we tested the response of DBL-1 variants to this larger anesthetic over time. We found that tricaine affects DBL-1 over- and under-expressing animals in manner similar to levamisole, where *dbl-1(++)* animals are resistant, while *dbl-1(nk3)* animals are more sensitive to tricaine-induced paralysis (Figure 2B). IP2P, a smaller anesthetic with a molecular weight of 152, acts as an anesthetic by eliminating neural activity and blocking muscular contraction [45]. We asked if DBL-1 variant animals would display altered sensitivity to this lower molecular weight anesthetic. We found that *dbl-1* mutant animals are more sensitive to IP2P than wild-type animals (Figure 2C). Notably, we discovered that long *dbl-1(++)* animals are as sensitive as wild-type animals to the paralyzing effects of IP2P (Figure 2C). Unlike the other anesthetics tested here, the nematode anesthetic sodium azide acts by inhibiting the electron transport chain [46]–[48]. Further differentiating these anesthetics, sodium azide is much smaller, having a molecular weight of 65. We asked if DBL-1 variant animals display an altered response to this low molecular weight anesthetic. While sodium azide is commonly used for imaging at doses of 10–25 mM, which anesthetizes nematodes quickly [49], [50], we chose a lower dose, 1 mM, to test for differences in sodium azide sensitivity in *dbl-1* under- and over-expressing strains in our 90-minute assay. Similar to their behavior on the other tested anesthetics, small *dbl-1(nk3)* animals display a more sensitive response to sodium azide (Figure 2D). Like the result with IP2P, we discovered that long *dbl-1(++)* and *lon-2(e678)* animals are at least as sensitive to the paralyzing effects of sodium azide as wild-type animals (Figure 2D and data not shown). These results show that loss of DBL-1 results in hypersensitivity to multiple drugs of varied size and mode of action, while animals with enhanced DBL-1 signaling are resistant to the higher molecular weight drugs tested in this study, levamisole and tricaine. This data suggests that a lower dose of DBL-1 decreases barrier function, allowing higher molecular weight molecules to access targets, and lower molecular weight molecules increased access to targets. Concordantly, a higher dose of DBL-1 pathway signaling increases barrier exclusion of higher molecular weight molecules, but does not further decrease access of smaller molecules to their targets.

To quantify these differences in drug responsiveness, we determined the dose response of DBL-1 variant animals compared to wild type using a range of levamisole concentrations. We found that *dbl-1* mutants are more sensitive to levamisole, displaying increased susceptibility ranging from 0.25 to 1 mM levamisole with a half-maximal effective concentration (EC_50_) of 0.23, compared to an EC_50_ of 0.56 for wild-type animals (Figure 2E). *dbl-1(++)* animals are more resistant in higher concentrations of levamisole, with an EC_50_ of 0.83 (Figure 2E). These results indicate changes in DBL-1 signaling levels produce significant differences in sensitivity to a broad range of anesthetic concentrations.

### DBL-1 is required for normal cuticular permeability

In nematodes, the cuticle and its underlying hypodermis form a permeability barrier that protects the animal from its environment, where molecules in the environment must first breach the cuticle and then the hypodermal membrane to pass this two-fold natural defense. This environmental barrier is strong enough that nematodes submerged in 6% glutaraldehyde, a high concentration of fixative, continue to move even after 7 hours [51]. However, drugs and dyes can breach this diffusion barrier if their size and polarity permit it [52]–[55].

Increased permeability is associated with drug hypersensitivity in *C. elegans* [24], [25]. Because our drug assay results are consistent with DBL-1 signaling changes altering the nematode's physical barrier, we asked if altered drug responsiveness in the DBL-1 variant backgrounds is caused by changes in cuticle integrity, rather than the hypodermal cell membrane. Reducing levels of DPY-13, a cuticular collagen that is involved in covalently cross-linking cuticular proteins, specifically increases the permeability of the cuticle, thereby allowing more anesthetic to pass through the cuticle and reach receptors [56], [57]. If the altered response to levamisole seen in DBL-1 variant animals is due to defects in cuticular permeability, while retaining normal hypodermal barrier function, reducing DPY-13 in the *dbl-1(++)* background should restore sensitivity to levamisole and possibly present an even more sensitized response to levamisole in *dbl-1(nk3)* animals lacking DBL-1 activity. If, however, the resistance to levamisole in DBL-1 variant animals is caused by changes in the hypodermal membrane barrier, then increased permeability of the cuticle should not affect levamisole response in *dbl-1* overexpressing animals.

We tested the effect of DPY-13 depletion on cuticular permeability using gene-specific RNA interference (RNAi). While we found DPY-13 depletion through RNAi did not significantly sensitize wild-type animals to 0.5 mM levamisole, we did find that *dpy-13* RNAi significantly affected anesthetic response in animals with increased or decreased DBL-1 signaling, suggesting an incomplete reduction of DPY-13 function by RNAi (Figure 2F). We found that reducing DPY-13 levels in long *dbl-1(++)* animals restored sensitivity to levamisole to wild-type response levels (Figure 2F). Further, reduction of DPY-13 function in *dbl-1(nk3)* animals resulted in a significant increase in levamisole-induced paralysis over time compared to *dbl-1(nk3)* animals (Figure 2F). These results show that DBL-1 signal perturbation sensitizes animals to the effects of reduced DPY-13 levels. This result is consistent with the model that DBL-1 affects the cuticle barrier rather than the hypodermal cell barrier. While DPY-13 affects cross-linkages within the cuticle, the enhanced sensitivity of *dbl-1(++)* or *dbl-1* mutant animals with reduced DPY-13 levels suggests that DBL-1 and DPY-13 act on different aspects of the nematode cuticle to control small molecule entry and therefore drug sensitivity. These genetic and pharmacological studies suggest that DBL-1 affects permeability of the cuticle, and therefore drug response, in a dose-dependent manner.

Because the finding that knockdown of DPY-13 specifically affects drug response in animals with abnormal DBL-1 levels suggests DBL-1 variants display cuticle defects, we directly tested cuticular permeability using Hoechst 33342, a DNA-binding dye with a molecular weight of 616 that fails to transpass the cuticle in wild-type animals, but stains nuclei in animals with an impaired cuticular surface barrier [58]. Wild-type control animals typically did not stain, nor did animals overexpressing DBL-1 (Figure 2G, I). However, animals lacking DBL-1 displayed increased staining within the population (Figure 2H, I), consistent with a role for DBL-1 in cuticular barrier function. To support our results, we tested *srf-5*, a gene whose product is required for normal cuticular surface properties, and found that loss of this gene product also increases cuticular permeability to Hoechst 33342 (Figure 2I).

### Loss of DBL-1 signaling causes worm-star aggregates in solution

**Figure 3:**
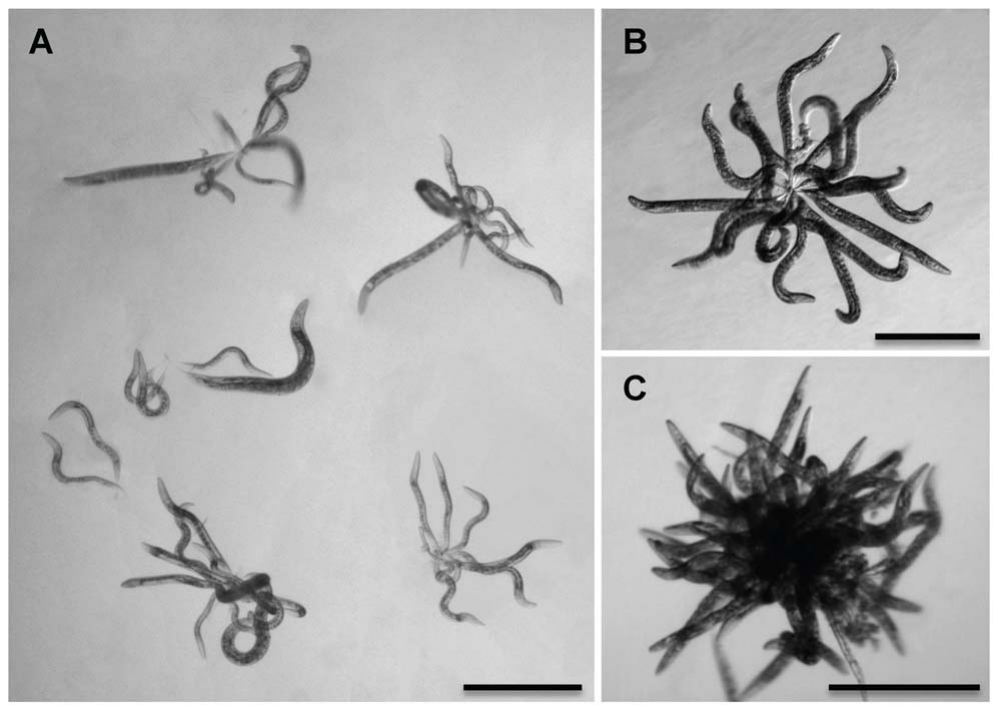
DBL-1 signaling affects surface adhesion. (A) Six worm-stars show larval and adult *dbl-1(nk3)* hermaphrodites knot by their tails. Scale bar  =  0.5 mm. (B, C) *dbl-1(nk3)* adult animals become tangled by their tails, forming moderate (B) or dense (C) worm-star aggregates in liquid. Scale bars  =  0.5 mm.

When the surface of *C. elegans* is coated with Verde1, a pathogenic, cuticle-adhering *Leucobacter* bacterial strain, animals can adhere to each other by their tail tips, creating an oriented aggregate [62]. This phenomenon, called “worm-stars”, has been previously described for multiple wild nematode species extracted from various sources (Table S1) [59]–[66]. Oriented clumps of animals were first described by Haut in 1956 as “spherical nematode-aggregates,” and have also been named “medusa-head formations,” “sunflowers,” or “rosettes.” Nematode aggregates can be disrupted by the addition of sodium bicarbonate, indicating surface ionic interactions promote tail entanglement [59], [67]. We found that both wild-type and *dbl-1(++)* animals did not form aggregates in liquid media lacking bacteria. However, *dbl-1(nk3)* animals formed worm-stars under similar conditions, approximately 10% of animals in an adult population (Figure 3). This incidence is lower than the incidence of worm-star formation in *C. elegans* populations in pathogenic bacteria. Juvenile hermaphrodites also formed clusters (Figure 3A). Worm-stars comprised as few as two animals with entwined tails (Figure 3A) to very densely populated aggregates (Figure 3B, C). Unlike the worm-stars that occur in the presence of pathogenic bacteria, *dbl-1(nk3)* worm-stars could be dissociated and typically did not cause the death of clumped animals (Figure 3 and data not shown). Animals lacking SMA-3, a transcription factor activated by DBL-1 receptor signaling, formed worm-stars with about 5% incidence (Figure S1), confirming that the worm-star phenotype is not specific to loss of DBL-1 function, but to DBL-1 pathway function.

**Figure 4:**
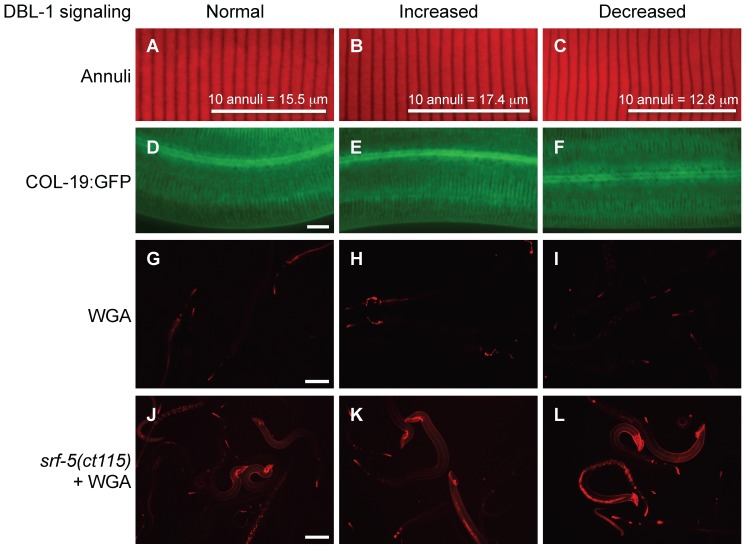
DBL-1 signaling affects specific cuticular surface properties. (A–C) Rhodamine-conjugated wheat germ agglutinin (WGA) staining in wild-type (A), *dbl-1(++)* (B), and *dbl-1(nk3)* (C) populations. Scale bar = 100 µm. (D–F) WGA staining in *him-5(e1490); srf-5(ct115)* animals with *C06C3.5(RNAi)* (pseudogene control RNAi) (D), *lon-2(RNAi)* (E), and *dbl-1(RNAi)* (F). Scale bar = 100 µm. (G–H) Staining of annuli in wild-type (G), *dbl-1(++)* (H), and *dbl-1(nk3)* (I) animals. Bars mark the length of 10 annuli and indicate the average length of 10 annuli for each strain. (J–L) COL-19:GFP expression in otherwise wild-type animals with *C06C3.5(RNAi)* (pseudogene control RNAi) (J), *lon-2(RNAi)* (K), and *dbl-1(RNAi)* (L). Scale bar = 10 µm.

Since animals lacking DBL-1 signaling display increased cuticular permeability and altered cuticular composition, we asked if loss of another gene known to regulate properties of the cuticular surface would also cause worm-star formation. To test this possibility, we used *srf-5(ct115)* mutant animals, which display altered surface antigenicity (see Figure 4D) [27], [68], increased cuticular permeability (Figure 2I), altered immune defense [68], [69], and have altered patterns of surface lipids [70]. A role for *srf-5* in susceptibility to infection is two-sided and dependent on the nature of the pathogen. For example, *srf-5(ct115)* mutant animals display increased resistance to a nematode-specific bacterium due to an inability of the pathogen to adhere to the cuticular surface [68]. However, *srf-5* mutant animals display increased susceptibility to nematophagous fungi [69]. Supporting the idea of altered surface properties causing aggregation, we found that adult hermaphrodites lacking functional SRF-5 formed worm-stars in liquid, with less than 5% of the hermaphrodites becoming entangled. Wild-type animals failed to aggregate in the same conditions (data not shown).

In populations of animals lacking DBL-1 pathway signaling or SRF-5, hermaphrodites of all stages were included in the oriented aggregates (Figure 3A and data not shown). To determine the requirement for the hermaphrodite and larval whip-like tail tip in worm-star aggregation, we used *sma-3(wk30)* and *srf-5(ct115)* strains that contain *him-5(e1490)*, which increases the incidence of males in the population. Interestingly, we found adult males, which display a specialized, blunt-ended tail structure instead of a tapered tail tip, were always excluded from these oriented aggregates. The one exception was a single *sma-3(wk30); him-5(e1490)* adult male that had not fully molted and was attached to a worm-star by its partially shed cuticle (Figure S1). The absence of adult males from these oriented aggregates suggests that tail whips contribute to worm-star formation in these mutant backgrounds.

Because worm-star formation is affected by altering the *C. elegans* surface coat [71]–[73], we asked if loss of the cuticle's surface coat would affect aggregation in wild-type animals. Previous studies in *C. elegans* have shown that treating wild-type animals with ethanol extracts surface lipids and removes their surface coat [70]. Indeed, we recapitulated the worm-star formation phenotype in wild-type animals using a 30% ethanol extraction, though less than 1% of the population was affected. Taken together, these results suggest alteration of cuticular surface properties in *dbl-1* and *srf-5* mutant animals permit worm-star formation. This is the first report of worm-star formation in mutant nematode backgrounds and shows that endogenous surface coat properties can be altered in wild-type *C. elegans* to promote aggregation.

### DBL-1 specifically affects ionic surface properties of the cuticle

Because our results indicate permeability of the cuticle and ionic surface properties are altered in DBL-1 variant strains, we asked if specific cuticular properties are altered in animals with decreased or increased DBL-1 signaling. The *C. elegans* cuticle is composed of different layers that entirely cover the animal's external surface in a flexible, resilient exoskeleton. This exoskeleton protects the animal from environmental insults and infection. A surface coat of charged glycoproteins, the glycocalyx, covers the epicuticle, a lipid-rich cuticular layer [70]. Under the epicuticle are the cortical, medial, and basal cuticular layers. Wheat germ agglutinin (WGA) binds glycoproteins and stains the *C. elegans* cuticular surface when surface antigenicity is altered, as in *srf* mutant animals [27], [37], [74], while the red fluorescent lipophilic dye DiI stains phospholipids [36], [75].

We first stained animals with reduced or overactive DBL-1 signaling relative to the wild type with fluorescently tagged WGA. DBL-1 variants were indistinguishable from the wild type, exhibiting limited staining, while positive control *srf-5* mutant animals stained extensively (Figure 4A-D). Abnormally exposed or accumulated mucin-type glycans bind WGA in *srf* mutants [74]. We asked if WGA binding protein is affected in DBL-1 variants when the lectin-binding protein is revealed by loss of SRF-5. We stained *him-5(e1490); srf-5(ct115)* animals RNAi-depleted of *dbl-1*, the DBL-1 inhibitor *lon-2*, or a pseudogene control with rhodamine-labeled WGA. Males, generated by *him-5(e1490)*, stain with WGA more robustly than hermaphrodites. WGA staining was like the *srf-5* background, again indistinguishable in all three DBL-1 signaling conditions (Figure 4D-F). This result shows DBL-1 does not affect surface antigenicity of WGA in *srf-5* mutant animals.

We then asked if DBL-1 pathway signaling affected external cuticle morphology. A previous report that analyzed DBL-1 pathway genes *sma-2*, a Smad transcriptional regulator, and *lon-2* showed a relationship between body length and distance between annuli, ridges patterning the cortical layer that ring the animal from nose to tail (see Figure 4G) [26]. Mild perturbations of annuli and longitudinal ridges called alae in *lon-2* mutant animals were noted [26]. Other mutations that affect cuticle and body length show differences in annular ridge width [21], [26]. Using DiI, a vital lipophilic dye that binds to lipids in the cuticular surface, to highlight annular furrows, we also found a direct correlation between annular width and body length in DBL-1 signaling variants (Figure 4G-I, Table 1) [36]. We did not discern noticeable aberrations in annuli or alae in animals with decreased or increased DBL-1 signaling (Figure 4G-I and data not shown).

Under the epicuticle lies the cortical layer of the cuticle. We asked if DBL-1 pathway signaling affected the distribution of a cortical cuticular component, COL-19 [26]. We could discern no significant differences in COL-19 organization between the wild type and RNAi knockdown of DBL-1 pathway members *dbl-1* and *lon-2*. This result is supported by previous observations of normal GFP-tagged COL-19 patterning in *sma-2* and *lon-2* mutant strains (Figure 4J-L) [26]. Together, these analyses suggest that the permeability and ionic surface defects of DBL-1 variants are not caused by gross defects in surface antigenicity or the patterning or organization of the cortical layer of the cuticle.

### DBL-1 pathway signaling regulates composition and organization of the cuticle

**Figure 5:**
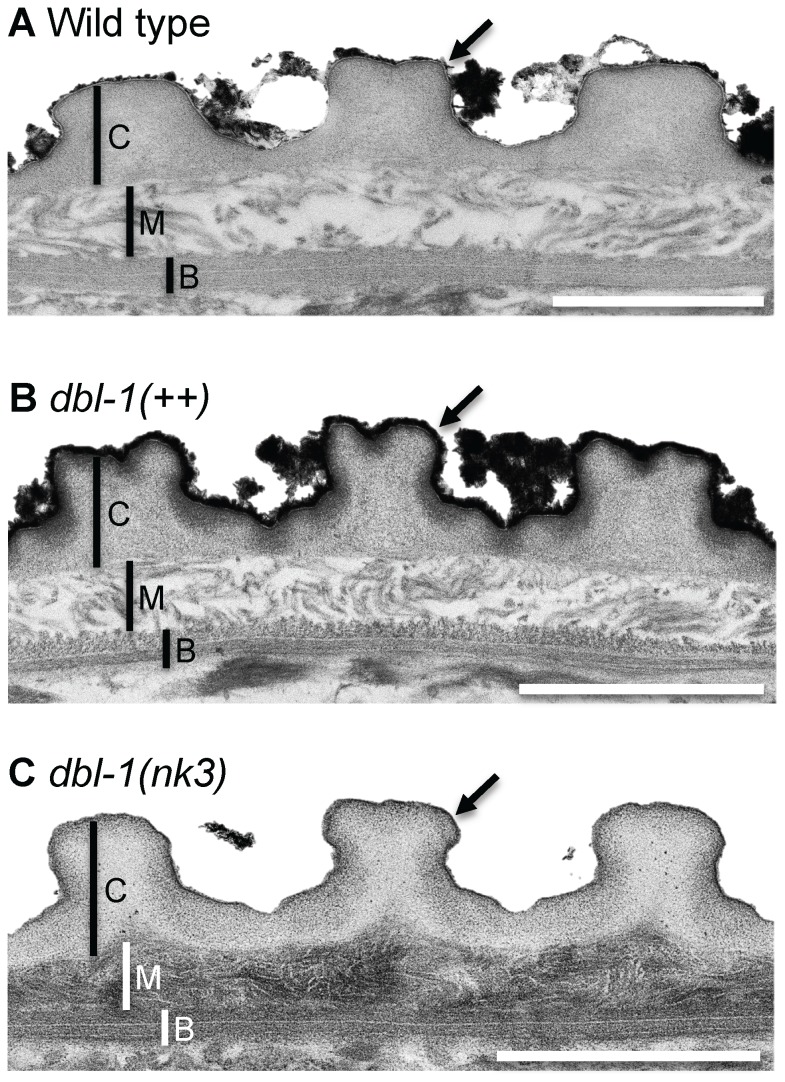
The DBL-1 pathway regulates cuticular organization and composition. Transmission electron microscopy (TEM) micrographs of wild-type (A), *dbl-1(++)* (B), and *dbl-1(nk3)* (C) animals. C indicates cortical layer; M indicates medial layer; B indicates basal layer; and the arrow marks the surface coat and epicuticular layer. Scale bars = 1 µm.

To identify the physiological basis of the DBL-1 dose-dependent response to anesthetics and body length and the worm-star phenotype displayed by animals deficient in DBL-1 signaling, we directly observed the cuticle of wild-type and DBL-1 signaling variant strains using transmission electron microscopy (TEM). We developed a microwave-assisted protocol that effectively and more quickly processes *C. elegans* specimens (see Materials and Methods) compared to traditional benchtop approaches. Because worm-star formation is only seen in wild-type animals that have had their outer lipid layer extracted, we also used malachite green, a classic dye used to preserve and stain lipids on the cuticle surface that would otherwise be extracted from samples during preparation [41]. This method differentiates the cuticular layers, which are thicker and readily distinguishable under the alae (Figure 5). We found that DBL-1 levels affect both the width and depth of the alae (Table 2). Further, phospholipids on the outer surface of the cuticle of wild-type animals are bound by malachite green (Figure 5A). This malachite green preservation of lipid was sensitive enough to reveal differences in the external surface of the cuticle that DiI staining could not resolve (Figure 4G-I). Long animals overexpressing DBL-1 have a thicker layer of malachite green staining the surface, suggesting an increased surface lipid content in this strain (Figure 5B). Consistent with the idea that animals deficient in DBL-1 pathway signaling display altered surface properties, small animals lacking DBL-1 have very little bound malachite green, indicating lipids are depleted on the outer surface of the cuticle in this background (Figure 5C).

**Table 2:** DBL-1 levels affect the dimension of cuticular components.

Genotype	Alae width (µm)	Alae depth (µm)	Cortical layer depth (µm)	Medial layer depth (µm)
Wild type	3.06±0.06	1.15±0.07	0.55±0.05	0.39±0.04
*dbl-1(++)*	2.41±0.10***	0.84±0.07*	0.51±0.03	0.22±0.03*
*dbl-1(nk3)*	2.48±0.12*	0.99±0.03*	0.63±0.06	0.21±0.01**

Comparison of cuticular components in animals with varying levels of DBL-1 signaling. Values indicate the mean ± SEM. P-values compare data to wild type (***P≤0.0001; **P≤0.001; *P≤0.05) using the unpaired t-test. n = 6 for each genotype.

Further, malachite green preserves and differentially stains the inner layers of cuticle, clearly distinguishing the collagen and cuticlin-containing cortical, fluid-filled medial, and oriented collagen fiber-formed basal layers (Figure 5). We found that the cortical layer is largely indistinguishable in both size and organization in wild-type, *dbl-1(++)*, and *dbl-1(nk3)* animals (Figure 5 and Table 2). Ultrastructural analysis revealed that DBL-1 also affects both the medial and basal layers of the cuticle. *dbl-1(nk3)* animals (Figure 5C) have a strikingly denser medial layer compared to wild-type (Figure 5A) and *dbl-1* overexpressing animals (Figure 5B). DBL-1 levels also affect the dimensions of the medial layer, where increased or decreased DBL-1 signaling is associated with a significant decrease in the depth of the medial layer (Table 2). The composition of the topmost basal sublayer in some animals overexpressing DBL-1 (Figure 5B) is less organized compared to either wild-type (Figure 5A) or *dbl-1(nk3)* animals (Figure 5C).

Alteration of both the external lipids and underlying cuticular layers suggests a physiological mechanism for the dose-dependent DBL-1-mediated drug responsiveness and body length phenotypes. These findings also suggest a mechanism for the Hoechst 33342 staining and worm-star formation phenotypes seen in animals lacking DBL-1. The reduced malachite green-stainable lipid layer may be important to prevent tail knotting. Reduction of this layer in *dbl-1* loss-of-function animals may increase surface adhesive properties by affecting surface ionic interactions.

## Conclusions

This work demonstrates that *C. elegans* DBL-1 shares a similar function with other BMPs in regulation of extracellular matrix. We provide a mechanism to largely explain some of the dose-dependent, seemingly disparate pleiotropic defects exhibited by DBL-1 pathway mutant animals [76], [77]. While previous work indicates the hypodermis is a main DBL-1 target tissue, we show that DBL-1 signaling targets cuticle, a specialized extracellular matrix secreted, at least in part, by the hypodermis. Whether these cuticle phenotypes, especially the lipid layer differences, are strictly hypodermally derived or if DBL-1 signaling affects different tissues will help clarify the organismal context of this cellular signaling pathway. Using drug assays and a novel genetic analysis, our data suggests that DBL-1 dose-dependent permeability alterations in the cuticle underlie the drug response phenotype. We assessed cuticular permeability directly with the dye Hoechst 33342 and found that *dbl-1* loss-of-function animals are more likely to stain with the nuclear dye Hoechst 33342 than the dye-impermeable wild-type and *dbl-1* over-expressing strains. Loss of DBL-1 facilitates tail entanglement and promotes worm-star formation, another novel phenotype we identified for *dbl-1* loss-of-function populations in *C. elegans*. Malachite green staining of lipids shows a reduced lipid layer coat in animals lacking DBL-1 signaling, which we propose underlies the worm-star aggregation phenotype of *dbl-1* signaling mutant animals. We identified dose-dependent DBL-1-mediated lipid changes and other ultrastructural composition and organization differences of the cuticle, which we propose are responsible for the DBL-1 drug response and body length phenotypes.

However, body length is a multifactorial phenotype that has other contributing factors, including endoreduplication, environment quality, and other signaling pathways, that may ultimately affect body length independent of cuticle [77].

The use of *C. elegans* as a tool for anthelmintic drug screening is a practical consideration, but the cuticle of *C. elegans* is more restrictive than some common parasitic nematodes [25], [78]. Using a *dbl-1* mutant background with its more permeable cuticle may prove useful for drug screening analyses.

Our studies also suggest a physiological mechanism for the increased susceptibility of DBL-1 pathway mutant animals to infection by bacteria and nematophagous fungus [79]–[83]. Not only is DBL-1 highly up-regulated in innate immune responses, but genes involved in an effective innate immune response are significantly up-regulated by DBL-1 signaling without significant differences in immune challenge [17]–[19], [79], [84]. Infection of DBL-1-deficient animals may be facilitated by the altered surface properties or compromised barrier function of the cuticle.

In addition, the male spicule, a rigid structure used to probe for and pry open the hermaphrodite vulva during mating, is crumpled in the *dbl-1* loss-of-function background [6], [85], [86]. Defective function of socket cells, which secrete the stiff cuticle that encases the spicule, is responsible for this phenotype [87]. While the *dbl-1* spicule defect is associated with mismigration of surrounding cells that mold the spicule cuticle, we propose that altered cuticle secreted by socket cells in *dbl-1* mutant animals contributes to the crumpled spicule defect.

**Figure 6:**
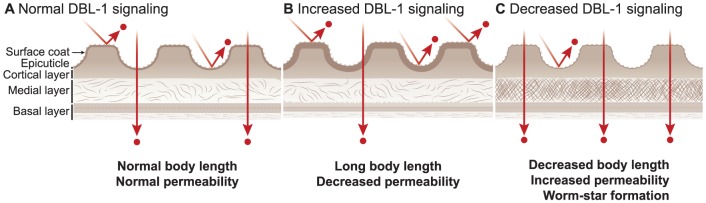
Model of DBL-1 pathway-mediated cuticular phenotypes. Model of how DBL-1 controls organization and composition of the cuticle, which affects body length, permeability barrier function, and worm-star formation in animals with normal (A), increased (B), and decreased (C) DBL-1 pathway signaling. Cuticle layers are indicated on the left.

We propose a model in which DBL-1 controls extracellular matrix organization and composition in a dose-dependent manner (Figure 6). In this model, decreased DBL-1 signal strength leads to changes in the cuticle that decrease body length and barrier function, leading to drug hypersensitivity, uptake of a normally cuticle-impermeable dye, and worm-star formation. Increased DBL-1 signaling causes cuticle changes that increase body length and barrier function, which results in drug insensitivity. The molecular tools we developed in the *in vivo* nematode model system may provide an attractive means to identify novel mechanisms and modulators of BMP signaling in extracellular matrix regulation.

## Supporting Information

Figure S1 **Unshed cuticle traps** ***sma-3*** **mutant adult male in a worm-star.** A rare instance of a *sma-3(wk30); him-5(e1490)* adult male (tail is indicated with a white arrowhead) entangled by its unshed cuticle (black arrowhead) in a worm-star (point of entanglement is marked with an arrow). Scale bar = 0.1 mm.
(TIFF)

Table S1 **Reports of aggregate formation in wild-type and mutant nematodes.** WT indicates wild-type populations.
(TIFF)
