# Correction: Regulation of Extracellular Matrix Organization by BMP Signaling in *Caenorhabditis elegans*

**DOI:** 10.1371/journal.pone.0118036

**Published:** 2015-01-30

**Authors:** The PLOS ONE Staff

There is an error in Fig. 4. Please see the corrected Fig. 4 here.

**Figure 4:**
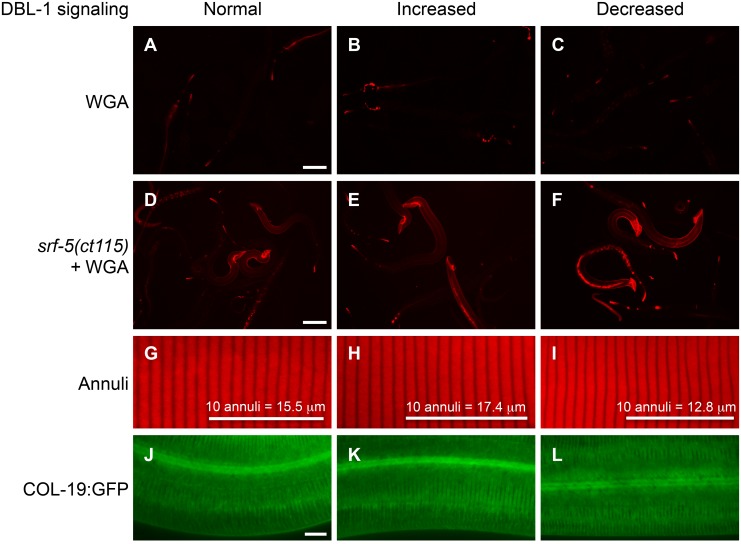
DBL-1 signaling affects specific cuticular surface properties. (A–C) Rhodamine-conjugated wheat germ agglutinin (WGA) staining in wild-type (A), dbl-1(++) (B), and dbl-1(nk3) (C) populations. Scale bar = 100 µm. (D–F) WGA staining in him-5(e1490); srf-5(ct115) animals with C06C3.5(RNAi) (pseudogene control RNAi) (D), lon-2(RNAi) (E), and dbl-1(RNAi) (F). Scale bar = 100 µm. (G–H) Staining of annuli in wild-type (G), dbl-1(++) (H), and dbl-1(nk3) (I) animals. Bars mark the length of 10 annuli and indicate the average length of 10 annuli for each strain. (J–L) COL-19:GFP expression in otherwise wild-type animals with C06C3.5(RNAi) (pseudogene control RNAi) (J), lon-2(RNAi) (K), and dbl-1(RNAi) (L). Scale bar = 10 µm.

There is information missing from S1 Table. Please view the correct S1 Table below.

## Supporting Information

S1 Table Reports of aggregate formation in wild-type and mutant nematodes. WT indicates wild-type populations.
(TIF)
