# Supplementary figures and images for: Regulation of Extracellular Matrix Organization by BMP Signaling in Caenorhabditis elegans
Source: PLoS One. 2014 Jul 11;9(7):e101929. doi: 10.1371/journal.pone.0101929 (PMC4094471; doi:10.1371/journal.pone.0101929)

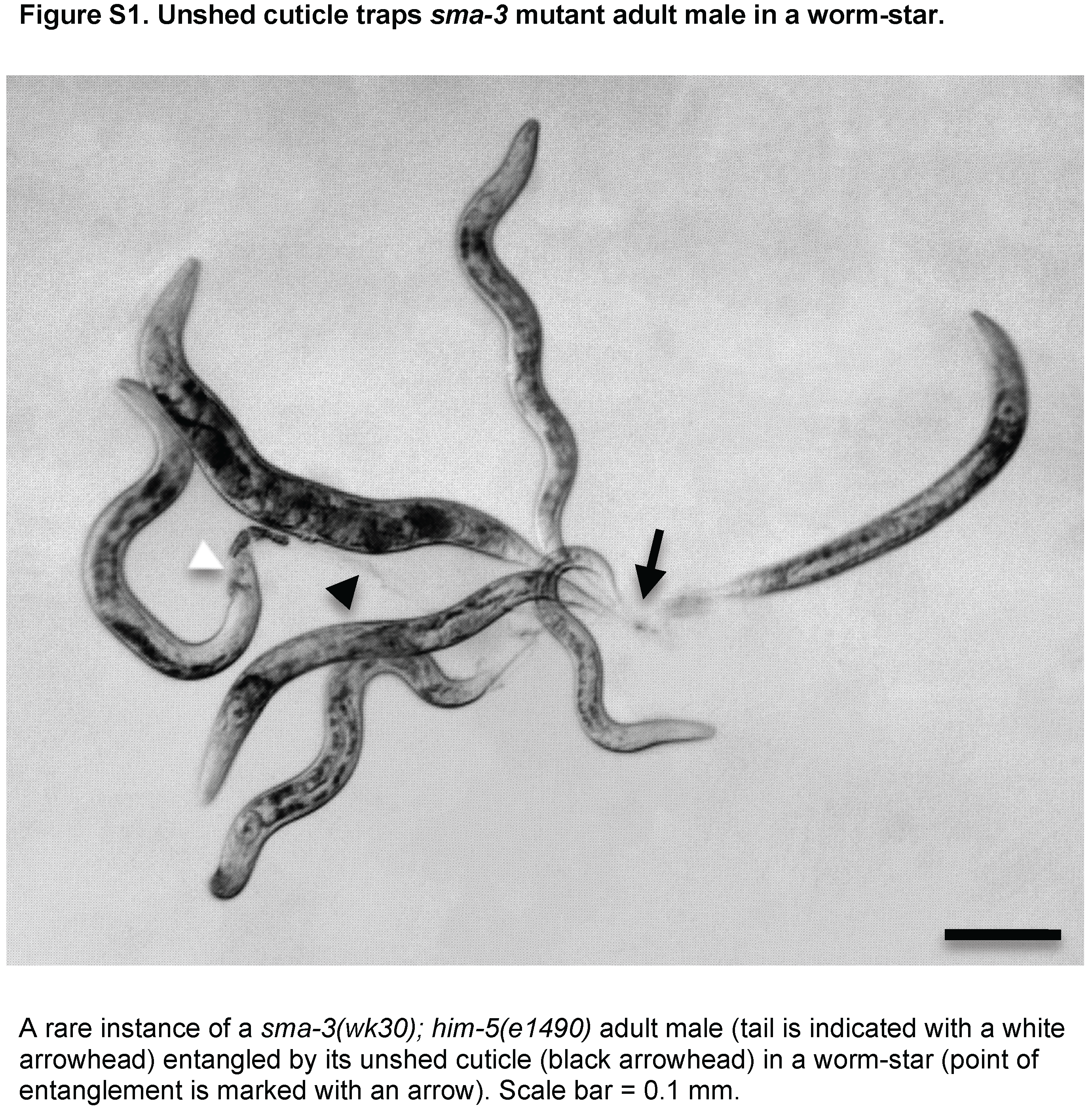

Supplement: Figure S1 — Unshed cuticle traps sma-3 mutant adult male in a worm-star. A rare instance of a sma-3(wk30); him-5(e1490) adult male (tail is indicated with a white arrowhead) entangled by its unshed cuticle (black arrowhead) in a worm-star (point of entanglement is marked with an arrow). Scale bar = 0.1 mm. (TIFF) [file pone.0101929.s001.tif]
